# Does clinical training improve mentalization skills in future therapists? A comparison of first and last year students of clinical psychology and of engineering

**DOI:** 10.3389/fpsyg.2023.1066154

**Published:** 2023-01-23

**Authors:** Steffen André Fagerbakk, Silje Helen Sørhøy, Torbjørn Nilsen, Nina Jakhelln Laugen

**Affiliations:** Department of Psychology, NTNU, Norwegian University of Science and Technology, Trondheim, Norway

**Keywords:** mentalization, reflective functioning, therapist skills, clinical psychology education, therapy training, students of clinical psychology, therapist characteristics

## Abstract

**Objective:**

Mentalization has been suggested as a therapist skill that might be important for therapeutic success. The aim of this study was to explore whether the mentalization capacity of students of clinical psychology differs from other students, and whether last-year students differ from first-year students.

**Method:**

A total of 297 students participated in this study, recruited from first and last years of psychology and engineering study programs. All participants filled out the MentS, a self-report measure of mentalization capacity.

**Results:**

No significant differences were found in self-reported mentalization capacity between first and last year students of clinical psychology. The results did however show that first year students of psychology had significantly higher self-reported mentalization skills compared to students of engineering, and higher MentS-scores were associated with gender (female) and higher age.

**Conclusion:**

The finding that students of clinical psychology rate themselves as having a higher capacity to mentalize compared to students of engineering might suggest that individuals with a higher capacity to mentalize are more likely to engage in clinical psychology and become therapists. However, the lack of significant difference in self-reported mentalization capacity in last year students of clinical psychology compared to first year students might indicate that the Norwegian education in clinical psychology does not succeed in enhancing mentalization in future therapists. Clinical psychology study programs might benefit from targeted mentalization training.

## Introduction

Before entering the clinical practice of their profession, students of clinical psychology must acquire not only theoretical expertise but also interpersonal skills in order to master the complicated interactions that often occur in therapy. Such interpersonal skills are found to potentially make therapists more effective at facilitating change in patients (Anderson et al., 2009; Heinonen and Nissen-Lie, 2020). Mentalization has been identified as one of these skills (Cologon et al., 2017), it influences the quality of interpersonal interactions and broadly refers to the ability to understand mental states that underlies behavior in oneself and others (Fonagy, 2008, p. 3). A study by Cologon et al. (2017) found that therapists with a higher capacity to mentalize generally achieved better therapy outcomes. If mentalization is a therapist characteristic that contributes to therapist effectiveness, universities should strive to enhance this capacity in students of clinical psychology. The current study aims to examine self-reported mentalization capacity in psychology students compared to students of engineering, and to investigate whether last year students of clinical psychology have a higher capacity for mentalization than first year students.

### Mentalization as a therapist skill

Research suggests that some therapists are more effective than others (Barkham et al., 2017; Johns et al., 2019), and that the most effective therapists on average are nearly twice as effective as the least effective therapists (Firth et al., 2015; Saxon et al., 2017, p. 712). Therapist effects account for approximately 3%–8% of the variability in therapy outcomes across a variety of therapy methods, therapy contexts, patient groups, outcome measures and study designs (Baldwin and Imel, 2013; Barkham et al., 2017; Johns et al., 2019). Whereas objective therapist characteristics such as age, gender, level of experience, theoretical orientation, and adherence to the treatment protocol do not seem to contribute significantly to variability in therapist effectiveness (Wampold et al., 2017; Heinonen and Nissen-Lie, 2020), interpersonal skills such as empathy, verbal and non-verbal communication skills and capacity to form constructive alliance bonds predict better therapy outcomes (Barkham et al., 2017; Heinonen and Nissen-Lie, 2020).

Mentalization can be defined as “the capacity to conceive of mental states as explanations of behavior in oneself and in others” (Fonagy, 2008, p. 3). This involves interpreting human behavior in terms of intentional mental states, such as needs, desires, feelings, beliefs, goals, purposes, and reasons. Therapists with highly developed mentalization skills will benefit from this in their performance of interpersonal skills, for example in the process of establishing a therapeutic alliance (Allen et al., 2008), and it is suggested that fostering mentalization has a decisive effect on all psychotherapeutic treatments and should be recognized as a common factor (Fonagy and Allison, 2012, p. 28; Allen, 2013). In a study including 25 therapists and 1,001 clients, therapists exhibiting a higher capacity to mentalize were more effective in terms of their therapy outcomes across patients. High mentalization capacity also compensated for the potentially harmful effects of an insecure anxious attachment style in therapists (Cologon et al., 2017). Similarly, Reading et al. (2019) found that therapists’ mentalization capacity was correlated with decrease in symptoms and interpersonal problems in patients measured at a 6-month follow-up after a brief relational therapy. Therapists’ mentalization capacity also predicted greater therapist-reported working alliance, greater patient reported depth, and greater patient and therapist reported degree of resolving ruptures. Also, in a study addressing problematic countertransference experiences in therapy with patients with borderline personality disorder, therapists who reported higher self-oriented mentalization skills also reported fewer problematic countertransference experiences (Bhola and Mehrotra, 2021). Specifically, higher levels of self-mentalization capacity were associated with lower likelihood of an over-concerned over-involved stance and parental feelings toward clients. Furthermore, mentalization has been found to have a small positive effect on therapists subjective well-being, and it was also found to mediate the negative effect of therapists’ insecure attachment on well-being (Brugnera et al., 2021). Thus, therapists’ mentalization capacity seems to impact therapy outcomes, therapy processes, and therapists’ well-being. For the same reasons, the present study will investigate mentalization in students of clinical psychology who aspire to become therapists.

### Mentalization and individual differences

There are individual differences in mentalization skills. These might be related to each individual’s development: specifically, mentalization is proposed to be closely connected to the attachment system (Fonagy et al., 2002, p. 40). Securely attached individuals display a superior capacity to mentalize compared to those with insecure, particularly disorganized patterns (Luyten and Fonagy, 2015, pp. 373–374; Luyten et al., 2020).

Both in clinical and non-clinical samples, females are reported to have a higher capacity to mentalize than males (Bouchard et al., 2008; Abu-Akel and Bo, 2013; Jessee et al., 2016; Dimitrijević et al., 2018; Pazzagli et al., 2018; Köber et al., 2019; Rosso, 2022). Similar results have been found in studies of constructs related to mentalization. For example, Brackett et al. (2004) reported significantly higher emotional intelligence among female college students than among their male counterparts, recruited from the same psychology program. Furthermore, there have been reported differences between students at different study programs. In a study of 351 college students, students of humanities (e.g., social science, medicine, and biology) had a more empathizing cognitive style compared to students of science (e.g., mathematics, engineering, physics, and chemistry), which had a more systemizing cognitive style, independently of gender effects (Focquaert et al., 2007). Therapists in training have also been reported to have higher levels of mentalization skills than the population in general (Klasen et al., 2019). Rogoff et al. (2021) found that psychologists reported higher capacity for self-mentalization than the general population as measured by the RFQ18, although this study did not find any significant differences in self-reported capacity for mentalizing others between therapists, patients with borderline personality disorder, and members of the general population, which is quite counterintuitive. The authors argue that both therapists and patients with borderline personality disorder might have some impairments and enhancements of dimensions of mentalization, rendering such labels as “expert” and “poor” mentalization simplistic. Namely, BPD patients are identified with enhanced emotional empathy combined with impaired cognitive empathy which might impact their mentalization, while therapists enhanced mentalizing capacities may be defined by their ability to inhibit the impact of other’s emotions on the self, which in turn might influence their mentalization of others. The authors also note some possible problems with the RFQ18, for example too similar items in the RFQ-other scale and that it might fail to discriminate cognitive and emotional dimensions.

In sum, these studies suggest that individual differences in mentalization skills might be evident before carrier choices are made, as suggested by the associations with gender (Bouchard et al., 2008; Abu-Akel and Bo, 2013; Jessee et al., 2016; Dimitrijević et al., 2018; Pazzagli et al., 2018; Köber et al., 2019; Rosso, 2022) and attachment style (Fonagy et al., 2002, p. 40; Luyten and Fonagy, 2015, pp. 373–374; Luyten et al., 2020), and it is therefore plausible that these differences influence students’ choice of study major as individuals with high mentalization capacity might be more likely to choose humanities or clinical studies.

### Stability and change in mentalization skills

Mentalization is viewed as having both “trait” and “state” features (Fonagy and Luyten, 2009; Vrouva et al., 2012; Luyten et al., 2020). Mentalizing abilities are thought to be established in childhood and has been found to be protective in the event of potentially traumatic experiences (Allen, 2018) and aggressive behavior later in life (Taubner et al., 2013), suggesting a trait-like component of mentalization. This can be supported by observations of stability of some features of mentalization, for example alexithymia (reflecting serious problems with internally based mentalizing) has been found to be stable in a large cohort of adults followed up for 10 years (Hiirola et al., 2017). Mentalization is also to large extent relationship specific, and controlled mentalizing tends to be inhibited with increasing arousal or stress (Luyten et al., 2019). Stability in mentalizing may thus coexist with fluctuations across contexts. Considerable fluctuations in mentalizing abilities have been extensively documented in patients with BPD (Fonagy and Luyten, 2016), in non-clinical samples (Luyten et al., 2019) and in a community sample where stress and arousal levels were experimentally manipulated (Nolte et al., 2013).

Although early development and gender might account for some of the variability in mentalization skills, these skills are also assumed to develop as a result of experience. According to Allen et al. (2008, p. 320), insufficient mentalization is usually not related to deficient abilities, but rather a failure to cultivate mentalization and utilize it in interpersonal interactions. Being open and curious about mental and affective states in oneself and others over time, should improve mentalization skills. The most obvious reason why people might improve their mentalization skills with age, would be that older people simply have more experience in mentalization. This assumption was supported by a study showing a positive correlation between therapists’ age and facilitating interpersonal skills, which is a skill set assumed to be related to mentalization. The authors suggest that professional-and life experience leads to better mastery of these skills (Anderson et al., 2009).

There is evidence that mentalization can be improved in patients through therapy, and some therapy methods such as mentalization based treatment and transference-focused psychotherapy are commonly used in therapy with patient groups characterized with deficient mentalization, such as borderline personality disorder, to promote mentalizing abilities in these patients (Kernberg et al., 2008; Goodman, 2013; Fischer-Kern et al., 2015; Fonagy and Bateman, 2019, pp. 103, 323; Luyten et al., 2020). The documented change in mentalization among these patients, renders the possibility that healthy individuals also could achieve enhancement of mentalization through training. In fact, a study by Ensink et al. (2013) found that students of clinical psychology improved their mentalization skills after completing a mentalization training program. The program involved training students to recognize and differentiate own mental and emotional reactions in therapy, to recognize how these reactions may influence ones understanding of the patient and how they may contribute to or block the therapeutic process. Traditional didactic training, on the other hand, did in fact result in lower mentalization capacity (Ensink et al., 2013). Other therapist factors and characteristics have also been found to improve on novice therapists by instruction, modeling, deliberate practice, feedback and supervision, especially self-efficacy, empathy, the ability to form alliance and deal with emotions, and consciousness about countertransference (for a review see Knox and Hill, 2021).

Given the assumption that mentalization skills also benefit from increased experience with interpersonal situations (Allen et al., 2008, p. 320; Anderson et al., 2009), we hypothesize that students in therapy training who receive not only didactic training but also gain practical experience in therapy-related situations, improve their mentalization abilities even in programs that do not target mentalization specifically. This will be addressed in the current study.

### Measuring mentalization

Mentalization can be assessed by different forms of measures, for example questionnaires, interviews and narrative coding systems, experimental/observational tasks and performance-based measures (Luyten et al., 2019). As the various forms of assessment have different qualities, they also hold some methodological challenges (for a discussion, see Dimitrijević et al., 2018). Interview-based assessment methods may provide accurate assessment of mentalization capacity; however, they are time-and resource consuming, thus limiting their use to smaller samples (Dimitrijević et al., 2018). For example, the Reflective Function Scale (RFS: Fonagy et al., 1998), which is recognized as the gold standard in mentalization measurement, is unsuitable for large-scale quantitative studies because of how much time and expertise it requires, and has in fact only been validated in some clinical studies (e.g., Müller et al., 2006; Bouchard et al., 2008; Fischer-Kern et al., 2010) Self-report measures do not demonstrate one’s capabilities as accurately as performance measures do. Moreover, it is possible that individuals with low mentalization capacity might also respond inaccurately in a self-report questionnaire, thus causing potential validity issues. Still, they are more feasible to administer to larger samples. There have been several attempts to create self-report measures of mentalization, but with limited reliability, validity, and applicability (Dimitrijević et al., 2018). One example is the Reflective Functioning Questionnaire (RFQ: Fonagy et al., 2016). It has been shown to correlate positively with related constructs such as empathy, mindfulness, and perspective-taking, and negatively with indexes of psychopathology, while also discriminating between persons with personality disorders and controls, and predicting attachment. However, it has some problems with internal consistency, its validity can be questioned as it seems to only assess some of the aspects of mentalization, and it is argued that it can only capture hypomentalizing but not likely the maladaptive forms of hypermentalizing (Fonagy et al., 2016; Dimitrijević et al., 2018; Müller et al., 2022). In addition, it has only been related to clinical phenomena (e.g., empathy and attachment) and has not established links with “mainstream” personality constructs (e.g., the Big Five). In recent years, Dimitrijević et al. (2018) have developed the Mentalization Scale (MentS), which is a 28-item self-report measure of mentalization. It was initially examined in samples of employed adults, university students and persons with borderline personality disorder and matched controls (Dimitrijević et al., 2018). Acceptable levels of internal consistency were reported, as well as a meaningful factor structure and correlations with expected factors such as gender, level of education, attachment, personality traits, emotional intelligence and empathy. Other studies have also found similar factor structure and levels of internal consistency (Đorđević and Đorđević, 2019; Benoit, 2020; Stanojević et al., 2020; Ahmadian and Ghamarani, 2021; Bhola and Mehrotra, 2021; Jańczak, 2021; Richter et al., 2021). Investigations of construct validity have found correlations with attachment style (Đorđević and Đorđević, 2019; Benoit, 2020; Stanojević et al., 2020; Jańczak, 2021), empathy (Ahmadian and Ghamarani, 2021; Jańczak, 2021), borderline features, emotional intelligence and personality traits (Jańczak, 2021) in the way that is according to previous knowledge about mentalization. Also, Richter et al. (2021) found the MentS to have a large positive correlation with the Reflective Function Scale (0.652, *p* = 0.000). Thus, self-report may be a feasible and adequate option in order to study mentalization skills in larger samples.

### Study objective

In sum, studies suggest that therapists’ capacity to mentalize might enhance therapeutic processes. Consequently, study programs for future therapists should promote the development of mentalization skills in their students. Two factors might influence the mentalization skills in psychology students: First, students in therapy training are more exposed to situations likely to promote mentalization, such as engaging in conversations with others about beliefs and emotions and guidance from experienced psychologists. Also, doing therapy training under supervision and discussing patients with supervising psychologist will likely enhance mentalization skills in students. Thus, it is likely that mentalization skills are enhanced during the training period considering the notion that mentalization skills will improve by increased experience with interpersonal situations, especially involving people with high mentalization skills such as supervising therapists (Allen et al., 2008, p. 320; Anderson et al., 2009). Second, individuals with advanced mentalization skills might be more likely to select clinical study majors, suggesting that therapists in training have good mentalization skills already at the start of their studies, which can be supported by studies showing higher levels of mentalization capacity in students in training (Klasen et al., 2019). The current study will examine these two hypotheses. First, differences in self-reported mentalization capacity among students of clinical psychology in their first and final year of study will be examined and compared to a control group of students of engineering. We hypothesize that last year students of clinical psychology report higher mentalization scores than first year students given that they have been exposed to more mentalizing activities, and that this difference is more profound than the difference between first and last year students of engineering. Such a difference would indicate that current therapist training succeeds to enhance the capacity for mentalization in future therapists.

Second, differences in mentalization capacity between first year students of psychology and first year students of engineering will be examined. We hypothesize that there is a difference in favor of psychology students. We base our hypothesis on the assumption that individuals with motivation and skill to mentalize are more likely to engage in the field of psychology. A difference in favor of engineering students would thus be unexpected.

## Methods

### Procedure and participants

The study sample consisted of students at the Norwegian University of Science and Technology (NTNU). Students in their first and last year of clinical psychology, and first year students of fundamental psychology, were recruited as the main sample. Clinical psychology is a 6-year degree that involves theoretical, empirical, and clinical study of the human mind and behavior. Students admitted to this course must also complete practical therapist training with guidance from experienced psychologists. Completion of the clinical psychology program leads to authorization as a clinical psychologist. Students are admitted to the program based on their grades from high school. No previous training or university education is required. The first 3 years of the study program consist mainly of didactic studies of different disciplines of psychology and preparatory clinical practice including activities such as role-playing and apprenticeship practicum. The second half of the study program consists mainly of supervised clinical training, first at the university outpatient clinics where they have two patients in therapy for 14 weeks each (one adult and one child/youth) and observe 6 additional therapies performed by other students, and later a 6-month practicum in ordinary healthcare under supervision from an experienced psychologist.

The study program for fundamental psychology involves theoretical and empirical study of the human mind and behavior, and do not result in an authorization for clinical practice. Students are admitted to the program based on their grades from high school. No previous training or university education is required. The study program consists of didactic studies. These psychology programs do however have fairly similar curriculums in their first year, and students in both programs will have similar level of university training at the admission. Thus, students of fundamental psychology were included to increase the sample size since they will be similar to the students of clinical psychology in terms of age and level of education (they are all undergraduates), and they will be taking almost the exact same courses the first year. For comparison, students in their first and fifth year of engineering (e.g., mathematics, computer science, physics, chemistry, and biotechnology) were recruited as controls. Students are admitted to these programs based on their grades from high school. No previous training or university education is required. These study programs are either undergraduate programs (bachelor degree) which can be followed by a 2 year graduate program (master’s degree) or a combined 5-year study program which leads to a master’s degree. These study programs were chosen as controls because they have no studies of human mind or behavior, and therefore we can expect no significant improvements of mentalization skills related to the education. There were no other criteria for inclusion or exclusion.

Participants were recruited in university lectures or online through student organizations e-mail lists or Facebook pages. The participants were briefed about the character of the study and the confidentiality of data and signed an informed consent form. All participants filled out the MentS and demographic data (age, gender, study program and years of study). As a compensation for their effort, the participants could take part in the drawing of 20 gift vouchers of NOK 250. The study was approved by the Regional Committees for Medical Research Ethics and the Norwegian Centre for Research Data. Altogether 297 participants were included in this study. Their distribution across gender, age, and study program are presented in Table 1.

**Table 1:** Sample descriptive statistics.

	First year fundamental psychology	First year clinical psychology	Sixth year clinical psychology	First year engineering	Fifth year engineering
*n*	34	43	57	109	54
Age, M (SD)	22.6 (2.35)	23.1 (2.36)	27.6 (1.98)	20.9 (1.40)	25.0 (1.14)
Male, %	11.76	18.60	26.32	55.96	51.85

### Measures

The Mentalization scale (Dimitrijević et al., 2018) consists of 28 items describing characteristics related to mentalization (e.g., item 21 *“I am often confused about my exact feelings,”* item 23 *“People tell me that I understand them and give them sound advice,”* and item 24 *“I have always been interested in why people behave in certain ways”*), and responses are given on a 5-point Likert scale, ranging from 1 (completely disagree) to 5 (completely agree). A sum score maps the total mentalizing abilities score, where higher score indicates higher capacity for mentalization. Total scores range from minimum 28 to maximum 140. Three subscales can be extracted: *Self-Related Mentalization* (8 items)*, Other-Related Mentalization* (10 items), and *Motivation to Mentalize* (10 items). Previous studies have reported consistent factor structure and good internal consistency ranging from 0.7 to 0.86 (Dimitrijević et al., 2018; Đorđević and Đorđević, 2019; Benoit, 2020; Stanojević et al., 2020; Bhola and Mehrotra, 2021; Jańczak, 2021; Richter et al., 2021). MentS was translated to Norwegian for the present study by two psychologists fluent in English. This translation was translated back to English by another psychologist who has English as the first language and also speaks fluent Norwegian. This version was compared to the original version to ensure that the content was successfully translated, and necessary adjustments were made. In the current study, the Norwegian version of MentS yielded good internal consistency measured by Cronbach’s α (total score: α = 0.88; subscales: α = 0.75–0.83).

### Statistical analyses

The data set was examined to ensure assumptions for the analyses were met, and adjustments were made accordingly. As the Norwegian version of MentS has not been previously used, preliminary tests were performed to provide an indication of its validity. A multiple linear regression was conducted to assess whether gender, study program, study year and age predicts total scores on the MentS in the direction previously described in the literature (e.g., Anderson et al., 2009; Abu-Akel and Bo, 2013; Dimitrijević et al., 2018). As the assumption of heteroscedasticity was not met, bootstrapping was used to generate confidence intervals and significance tests.

Independent samples t-tests were used to explore differences among first year students and last year students, for students of clinical psychology and for students of engineering separately. Tests were carried out for the MentS-Total and each of its three subscales. Students of fundamental psychology were excluded from these analyses as only first year students from this study program were measured. Cohen’s *d* was calculated as a measure of effect size.

One-way between groups analysis of variance (ANOVA) was carried out for the MentS-Total and each of the three subscales in order to explore group differences among first year students of clinical psychology, fundamental psychology and engineering. As MentS-Total and MentS-Motivation violated the assumption of homogeneity of variances, we report Welch’s statistic for these analyses. Furthermore, Games-Howell *post-hoc* test was selected because it can be liberal with small sample sizes and accurate when sample sizes are unequal (Field, 2013, p. 459).

## Results

### Preliminary analyses

Regression analysis showed that female gender, psychology study program, and higher age each contributed independently to higher MentS scores (Table 2). These three variables explained 26% of the observed variance in the total score.

**Table 2:** Summary of regression analysis for variables predicting MentS-total.

Variable	*B*	SE B	β	*p*	*R* ^2^	∆R^2^
Model 1					0.08	0.08
Constant	113.97 (110.29, 117.43)	1.88		0.001		
Gender	−6.51 (−9.14, −3.74)	1.35	−0.27	0.001		
Model 2					0.22	0.15
Constant	95.36 (89.14, 101.53)	3.12		0.001		
Gender	−3.09 (−5.78, −0.33)	1.33	−0.13	0.018		
Study program	9.55 (7.15, 11.96)	1.24	0.41	0.001		
Model 3					0.24	0.02
Constant	91.26 (84.55, 98.14)	3.19		0.001		
Gender	−3.2 (−5.94, −0.42)	1.30	−0.14	0.013		
Study program	9.18 (6.74, 11.64)	1.25	0.39	0.001		
Study year	3.49 (0.95, 5.91)	1.24	0.15	0.008		
Model 4					0.26	0.02
Constant	81.91 (70.95, 92.33)	5.27		0.001		
Gender	−3.53 (−6.16, −0,83)	1.29	−0.16	0.007		
Study program	7.51 (4.14, 10.42)	1.54	0.32	0.001		
Study year	0.38 (−3.43, 3.54)	1.83	0.02	0.84		
Age	0.7 (0.15, 1.37)	0.33	0.19	0.025		

### Differences in MentS scores among first and last year students

Descriptive statistics and mean differences between first year students and last year students are displayed in Table 3. There were no significant differences between first and last year students of clinical psychology nor engineering students for MentS scores. In fact, only the difference between first and last year students of clinical psychology in the self-subscale was close to the significance threshold. However, Cohen’s *d* confirmed that all group differences only represented small effects.

**Table 3:** *T*-test for comparisons of first and last year students.

	Descriptive statistic			*t*-test					
	*n*	*M*	SE	*t*	df	Mean difference	CI 95%	*p*	*d*
**MentS-total**									
Clinical psychology				0.48	98	0.66	−2.11, 3.43	0.636	0.01
First year students	43	112.44	1.05						
Last year students	57	113.10	0.92						
Engineering				1.43	161	2.74	−1.04, 6.52	0.154	0.23
First year students	109	99.22	1.04						
Last year students	54	101.96	1.74						
**MentS-self**									
Clinical psychology				1.94	98	1.24	−0.03, 2.50	0.056	0.23
First year students	43	31.33	0.54						
Last year students	57	32.56	0.38						
Engineering				1.13	161	0.95	−0.71, 2.61	0.259	0.19
First year students	109	28.77	0.49						
Last year students	54	29.72	0.65						
**MentS-other**									
Clinical psychology				0.19	98	0.13	−1.20, 1.46	0.847	0.04
First year students	43	40.19	0.53						
Last year students	57	40.32	0.43						
Engineering				1.36	161	1.12	−0.51, 2.76	0.177	0.22
First year students	109	36.49	0.47						
Last year students	54	37.61	0.70						
**MentS-motivation**									
Clinical psychology				−1.23	98	−0.69	−1.80, 0.42	0.223	0.25
First year students	43	40.93	0.37						
Last year students	57	40.24	0.40						
Engineering				0.78	161	0.66	−1.02, 2.35	0.438	0.13
First year students	109	33.97	0.49						
Last year students	54	34.63	0.71						

Cohen’s d small effect = 0.2, medium effect = 0.5, large effect = 0.8 (Cohen, 1992).

### Differences in MentS scores among first year students

MentS scores among first year students are displayed in Table 4. ANOVAs yielded significant group differences for the total MentS and for its three subscales. Large effect sizes were found for the MentS-total and the Motivation-subscale, medium to large effect for the Other-subscale and small to medium effect for the Self-subscale (Kirk, 1996).

**Table 4:** ANOVA by study group first year students.

	Descriptive Statistics				ANOVA			
	*n*	*M*	SD	CI 95%	df	*F*	*p*	ω^2^
**MentS-total**					2, 80.52^a^	40.06^a^	<0.001	0.23
Engineering	109	99.22	10.83	97.17, 101.28				
Fundamental Psychology	34	104.62	10.51	100.95, 108.29				
Clinical Psychology	43	112.44	6.87	110.33, 114.56				
**MentS-self**					2, 183	5.34	0.006	0.05
Engineering	109	28.77	5.18	27.79, 29.75				
Fundamental Psychology	34	28.32	4.52	26.75, 29.90				
Clinical Psychology	43	31.33	3.54	30.24, 32.42				
**MentS-other**					2, 183	10.92	<0.001	0.10
Engineering	109	36.49	4.87	35.56, 37.41				
Fundamental Psychology	34	38.41	4.45	36.86, 39.96				
Clinical Psychology	43	40.19	3.45	39.13, 41.25				
**MentS-motivation**					2, 84.26^a^	63.94^a^	<0.001	0.30
Engineering	109	33.97	5.10	32.99, 34.93				
Fundamental Psychology	34	37.88	4.26	36.40, 39.37				
Clinical Psychology	43	40.93	2.44	40.18, 41.68				

ω^2^ = omega squared effect sizes: small effect = 0.01, medium effect = 0.06, large effect = 0.14 (Kirk, 1996, p. 751).

a Welch’s statistic.

Games-Howell *post-hoc* analysis confirmed that first year students of clinical psychology scored higher than students of fundamental psychology and students of engineering on both the total MentS and its three subscales. Specifically, students of clinical psychology had a significantly higher total score than students of fundamental psychology (*p* = 0.001, *d* = 0.88) and students of engineering (*p* ≤ 0.001, *d* = 1.46). Furthermore, students of clinical psychology scored significantly higher on the Self-and the Motivation-subscale, compared to both fundamental psychology (Self: *p* = 0.006, *d* = 0.74, Motivation: *p* = 0.001, *d* = 0.88) and engineering (Self: *p* = 0.002, *d* = 0.58, Motivation: *p* ≤ 0.001, *d* = 0.17). For the Other-subscale, only the difference between clinical psychology and engineering reached significance (*p* ≤ 0.001, *d* = 0.88), indicating that students of clinical psychology scored higher on other-related mentalization compared to engineering, but not compared to fundamental psychology (*p* = 0.143, *d* = 0.45).

As for students of fundamental psychology, they also had a significantly higher total score compared to students of engineering (*p* = 0.032, *d* = 0.51) as well as on the Motivation-subscale (*p* ≤ 0.001, *d* = 0.83). However, differences did not reach significance for neither the Self-subscale (*p* = 0.879, *d* = 0.09) nor the Other-subscale (*p* = 0.088, *d* = 0.41).

## Discussion

This study aimed to contribute to the line of research that has identified mentalization as a potentially important therapist skill, by exploring the mentalization abilities among students of clinical psychology at the beginning and the end of their education, compared to students of engineering. The first hypothesis was that last year students of clinical psychology would have a higher self-reported capacity to mentalize compared to first year students. However, this hypothesis was not supported as the results showed no significant difference between these groups. The second hypothesis was that first year psychology students would have a higher self-reported capacity to mentalize compared to first year engineering students. The results showed that psychology students scored significantly higher on the total MentS and its three subscales compared to engineering students, thus confirming the second hypothesis.

The Norwegian version of the MentS indicated satisfactory internal consistency. Moreover, females scored significantly higher than males and higher age predicted higher MentS scores. These results are in line with expectations based on previous research (Anderson et al., 2009; Abu-Akel and Bo, 2013; Dimitrijević et al., 2018).

### Differences between first year students and last year students

Our study found no significant differences in self-reported mentalization capacity between first and last year students, neither among students of clinical psychology nor students of engineering. The assumption was that years of clinical training contributes to enhancement of mentalization capacity, and an improvement among students of clinical psychology was therefore expected.

It is reasonable to expect that students of clinical psychology would have a good basis for improving their mentalization capacity considering that they have high self-reported mentalization capacity at the beginning of their education. Previous studies have found that mentalization capacity can be improved through therapy (Fonagy and Bateman, 2019, pp. 103, 323; Luyten et al., 2020) and Ensink et al. (2013) found that psychology students significantly improved their mentalization capacity after attending a mentalization course. Their findings show that specific training in mentalization skills can make positive change in a relative short time span. They also observed a decrease in mentalization skills for a control group who only had didactic training and no training in mentalization. This indicates that the capacity for mentalization does not develop spontaneously through clinical training but must be targeted specifically. These results may also render the possibility that a narrow focus on diagnostic criteria and formulations of treatment plans might cause a decline in mentalization capacity. Although knowledge about diagnostic criteria and treatment plans are essential in clinical practice, one should be aware of the potential downfalls with excluding targeted mentalization training in the curriculum.

Another possible explanation for our results is that self-report measures might not successfully measure actual mentalization due to response bias. Self-report measures are based on participants self-evaluation, and not objective observations of actual behavior in interpersonal interactions (e.g., Murphy and Lilienfeld, 2019). Thus, participants might not have an accurate experience of their own mentalization capacity, it can be distorted by characteristics in the person such as self-confidence. Also, clinical education and training might provide students of clinical psychology with more insight about what it is possible to know about themselves and others. The clinical program does include both self-reflection and training in critical thinking about science. Therefore, students of psychology might become more critical about their own knowledge and about what they can know about themselves and others. Thus, the effect of growing critical thinking might confound the effect of growing mentalization skills in psychology students. Furthermore, social desirability might be a confounder of the student’s responses as mentalization is viewed as a desirable quality for a psychologist. This might lead to systematic bias in the students’ responses to the MentS. Also, it might be possible that students who are admitted to the clinical program gain a new perspective on themselves and view themselves as future psychologist. This new perspective might make them overconfident about their mentalization skills, leading them to report mentalization capacity in an overconfident way. Moreover, it is reasonable to assume that low mentalization skills involves less precise evaluation of these skills in the self, considering that these individuals often have a lack of interest in the mental world of themselves and others (Fonagy and Target, 2008; Luyten et al., 2019, pp. 39–43). This might lead to systematic bias in self-reporting by individuals with low mentalization skills.

The regression analysis showed a positive relationship between MentS-scores and age, but not with years of study. This differs from the findings of Dimitrijević et al. (2018), who found that higher education, and not age, was related to higher score on the MentS. Considering the hypothesis that capacity for mentalization improves simply by mentalizing (Allen et al., 2008, p. 320), especially in interpersonal contexts with individuals that possesses high mentalization skills, the capacity for mentalization should improve with age (Luyten et al., 2020). If this is true, then older people would have better mentalization skills simply because they have had more time practicing the skill. There are however few known studies that investigates the nature of age-related changes in mentalization skills. Conclusions regarding this are therefore yet to be made.

### Differences between first year students of psychology and engineering

First year students of clinical psychology scored significantly higher than engineering students on both the total MentS and each of the three subscales. Interestingly, students of clinical psychology also scored significantly higher than students of fundamental psychology for both the total MentS and the Self-and Motivation-subscales, but not for the Other-subscale. These results suggest that students of clinical psychology have a high self-reported capacity to mentalize already at the beginning of their education.

The preliminary hypothesis was that first year students of psychology would have a higher capacity to mentalize compared to engineering students. The assumption was that individuals with an motivation and skill to mentalize, would be more likely to select psychology as their major. Thus, differences in mentalization abilities should be evident even at the beginning of the course, and before students receive any therapist training. The field of psychology involves attempts to understand the interactions between the human mind and behavior, and the contextual environment in which it takes place, whereas mentalization broadly refers to the ability to understand mental states that underlies behavior in oneself and others (Fonagy, 2008, p. 3). It can be assumed that individuals who have a lower innate motivation and skill to engage in such activities should thus be less likely to engage in the field of psychology if choices are influenced by their levels of interests in the human mind and behavior.

The findings that first year students of psychology rate themselves as having higher capacity to mentalize compared to first year students of engineering support the assumption that mentalization capacity may predict student’s selection of study major to some extent. These results correspond with Focquaert et al. (2007) who found that students of humanities (e.g., social science, medicine, and biology) had a more empathizing cognitive style than students of science (e.g., mathematics, engineering, physics, and chemistry).

The significant differences between students of clinical psychology and fundamental psychology were not expected. The preliminary assumption was that students selecting any psychology program would possess similar levels of mentalization by the beginning of their studies. The admission process is based on high-school grades and does not involve any screening of their therapeutic abilities, such as the capacity to mentalize. Whereas fundamental psychology is concerned with the theoretical and empirical study of the human mind and behavior, clinical psychology also involves implementing this knowledge into direct interpersonal interactions with patients in therapy. This difference in course curriculum thus renders the possibility that students with a higher motivation and skill to mentalize, might be more likely to select the clinical perspective.

Among the psychology students in our study, there was an overrepresentation of female students. This rendered the possibility that the group differences in mentalization capacity were due to gender rather than study program. However, the regression analysis confirmed that study program significantly predicted total MentS scores even when accounting for the significant effects of gender. These findings strengthen the conclusion that students of psychology had a significantly higher self-reported capacity to mentalize than students of engineering, regardless of their gender. Why women seem to have better mentalization skills than males are not fully understood. A common stereotype is that women generally talk more about thoughts and feelings (i.e., mental states) than males do. This stereotype is supported by studies indicating that women are more emotional and emotionally expressive than are men (Brody and Hall, 2008). It also might be supported by research showing gender differences in constructs closely related to mentalization and attentiveness toward mental states. Evidence in current literature suggests that females have higher emotional intelligence compared to males, and that females generally have a more empathizing cognitive style (Brackett et al., 2004; Focquaert et al., 2007). Also, some studies have found that parents have more emotional content in their talk with daughters than with sons (Fivush et al., 2000; Aznar and Tenenbaum, 2015), and it is suggested that children are shaped into this gender difference in emotion expression by their parents and by their particular social environment (Chaplin, 2015). Luyten et al. (2020) argues that the capacity to mentalize is relationship-and context dependent, as mentalization develops in the context of interpersonal interactions and is continuously influenced by the mentalizing capacity of those who partake in these interactions. If females in fact do engage in more talk about mental states, and thus practice their mentalizing skills more than males, then this might be a possible reason why females have a higher capacity to mentalize. However, considering the common view that women are more emotional and emotionally expressive than men (Brody and Hall, 2008), it might be the case that women have an advantage in interview-based measures of mentalization because of their superior ability to express emotions. This view is supported by studies showing that women are more elaborate when depicting internal states (e.g., Fivush and Haden, 2003; Fivush et al., 2012; Grysman, 2018).

### Limitations and future research

This study presents new and interesting findings about the mentalization capacity among students of clinical psychology. However, some limitations of this study need to be addressed. The study uses a cross-sectional design, which may not be suitable to evaluate actual changes in mentalization capacity. Longitudinal designs represent a better basis for evaluating the development of individual mentalization skills, and may allow for within-subject comparisons of mentalization skills development in addition to between-subject comparisons. However, longitudinal designs are both time and resource demanding and were therefore beyond the scope of this study. Also, this study used voluntary response sampling and there is no information on those that were invited to participate but did not respond. This might be an issue in terms of representativeness, as the respondents might for example be those who were most interested in the field of study, leading to sampling bias. Moreover, there is need for more understanding of how mentalization develops in training programs as we currently do not know exactly what promotes mentalization and what does not.

As described throughout this discussion, there are some obvious limitations with the use of self-report measures. However, self-report measures are less time and resource demanding, and thus more suitable for use in larger samples. Future research should include additional measures for evaluation and assessment of the psychometric properties of the MentS. Assessments of the MentS reliability should include comparisons with established measures of mentalization, such as questionnaires (e.g., RFQ: Fonagy et al., 2016) and interviews (e.g., AAI: George et al., 1996). Because self-evaluations of one’s own mentalization capacity might be inaccurate and biased by individual characteristics, it could be advantageous to collect information from family, significant others, peers, and co-workers. Thus, it might be of interest to develop a version of the MentS for other-evaluation of mentalization capacity. Comparisons of self-evaluations vs. other-evaluations might contribute to more accurate assessment of mentalization. Other-evaluations of participants mentalization capacity might provide researchers with information about how individuals mentalization is experienced by others in interpersonal interactions. This would make sense considering that mentalization is a relational concept, and measurement should capture relational contexts.

The validity of self-report measures of mentalization is challenging to assess due to the complexity of the construct. Mentalization overlaps with other constructs that are different, but closely related to mentalization (e.g., empathy, theory of mind, and emotional intelligence). It can be challenging to determine if a self-report really measures mentalization and not related constructs. Because mentalization is a complex construct that involves several aspects of human behavior and mental states, self-report measures might not be sufficient for measuring all of these aspects.

The distribution of MentS-scores showed a lower spread among psychology students compared to students of engineering. This could suggest a ceiling effect, meaning that the MentS might not be sensitive enough for groups who have high mentalization skills. The assessment of mentalization in homogenous groups such as therapists might require different methods of measures than groups that are more heterogenous in terms of mentalization capacity. Furthermore, the MentS does not measure mentalization in terms of context or interpersonal relationships which rises some uncertainty about whether or not it can capture the “state” aspects of mentalization, considering it is assumed to be invariant across different relationships and contexts (Fonagy and Luyten, 2009). Other measures, such as the Reflective Functioning Scale as scored on the Adult Attachment Interview, involve the aggregation of mentalization across a number of attachment relationships.

## Conclusion

The current study explored the mentalization capacity of students of clinical psychology, using the self-report measure MentS (Dimitrijević et al., 2018). The results show that students of clinical psychology rate themselves as having a higher capacity to mentalize compared to students of engineering. This suggests that individuals with a higher capacity to mentalize are more likely to engage in clinical psychology and become therapists. If future research succeeds to establish mentalization skills as a common characteristic of effective therapists, the results of our study are promising. Furthermore, a motivation and skill to mentalize might constitute a significant factor in students possibility to enhance their mentalization capacity, as engaging in mentalizing activities have been suggested to be an important way to improve mentalization (Allen et al., 2008, p. 320; Ensink et al., 2013).

The current study did however not find any significant difference in self-reported mentalization capacity in last year students of clinical psychology compared to first year students. These results might indicate that current therapist training does not succeed in enhancing mentalization in future therapists. Thus, it is possible that specific mentalization training should be included in the curriculum, as such training have shown promising results in previous research (Ensink et al., 2013). However, future research should explore the development of mentalization in future therapists using longitudinal designs and other validated measures of mentalization.

There is a lack of validated measures that is easy to administer and that have low demands for time and resources. The MentS seems to be a promising instrument that might be a supplement to future research of individual differences in large samples. The current study confirmed the internal consistency and correlation with gender as reported in Dimitrijević et al. (2018). However, its validity is still uncertain, especially for groups who are expected to have high mentalization skills, such as therapists or students in therapist training. Future research should continue to assess the psychometric properties of the MentS by comparing individual MentS-scores with other validated measures of mentalization, and by including measures of other variables related to mentalization capacity (e.g., Fonagy and Bateman, 2006; Allen et al., 2017; Dimitrijević et al., 2018).

## Data availability statement

The raw data supporting the conclusions of this article will be made available by the authors, without undue reservation.

## Ethics statement

The study was approved by the Regional Committees for Medical Research Ethics (reference no. 62628) and the Norwegian Centre for Research Data (reference no. 212741). The patients/participants provided their written informed consent to participate in this study.

## Author contributions

SF and SS contributed to the study conception, design, material preparation, and data collection and analysis. NL and TN contributed to the literature search, analysis, and continuous revision of the manuscript. All authors contributed to the article and approved the submitted version.

## Conflict of interest

The authors declare that the research was conducted in the absence of any commercial or financial relationships that could be construed as a potential conflict of interest.

## Publisher’s note

All claims expressed in this article are solely those of the authors and do not necessarily represent those of their affiliated organizations, or those of the publisher, the editors and the reviewers. Any product that may be evaluated in this article, or claim that may be made by its manufacturer, is not guaranteed or endorsed by the publisher.

## References

[ref1] Abu-Akel A. Bo S. (2013). Superior mentalizing abilities of female patients with schizophrenia. Psychiatry Res. 210, 794–799. doi: 10.1016/j.psychres.2013.09.013, PMID: 24103909

[ref2] Ahmadian Z. Ghamarani A. (2021). Reliability and validity of Persian version of mentalization scale in university students. J. Fundament. Mental Health 23, 233–240. doi: 10.22038/JFMH.2021.18969

[ref3] Allen J. G. (2013). Mentalizing in the development and treatment of attachment trauma. 1st Edn. London: Routledge.

[ref4] Allen J. G. (2018). Mentalizing in the development and treatment of attachment trauma. London: Routledge.

[ref5] Allen J. G. Fonagy P. Bateman A. W. (2008). Mentalizing in clinical practice Arlington: American Psychiatric Publishing.

[ref6] Allen T. A. Rueter A. R. Abram S. V. Brown J. S. Deyoung C. G. (2017). Personality and neural correlates of Mentalizing ability. Eur. J. Pers. 31, 599–613. doi: 10.1002/per.2133, PMID: 29610548 PMC5877481

[ref7] Anderson T. Ogles B. M. Patterson C. L. Lambert M. J. Vermeersch D. A. (2009). Therapist effects: facilitative interpersonal skills as a predictor of therapist success. J. Clin. Psychol. 65, 755–768. doi: 10.1002/jclp.20583, PMID: 19437509

[ref8] Aznar A. Tenenbaum H. R. (2015). Gender and age differences in parent–child emotion talk. Br. J. Dev. Psychol. 33, 148–155. doi: 10.1111/bjdp.12069, PMID: 25387786

[ref9] Baldwin S. A. Imel Z. E. (2013). “Therapist effects: findings and methods” in Bergin and Garfield’s handbook of psychotherapy and behavior change. ed. Lambert M. J., vol. 6 (New Jersey: John Wiley & Sons), 258–297.

[ref10] Barkham M. Lutz W. Lambert M. J. Saxon D. (2017). “Therapist effects, effective therapists, and the law of variability” in How and why are some therapists better than others?: Understanding therapist effects. eds. Barkham M. Lutz W. Castonguay L. G.. eds. Castonguay L. G. Hill C. E. (Washington DC: American Psychological Association), 13–36.

[ref11] Benoit A. M. (2020). Examining relationships between early childcare Teachers' adult attachment orientations and quality of interaction in the infant classroom. Doctoral dissertation. Louisiana State University and Agricultural & Mechanical College]. LSU Digital Commons. Available at: https://digitalcommons.lsu.edu/gradschool_dissertations/5245

[ref12] Bhola P. Mehrotra K. (2021). Associations between countertransference reactions towards patients with borderline personality disorder and therapist experience levels and mentalization ability. Trends Psychiatry Psychother. 43, 116–125. doi: 10.47626/2237-6089-2020-0025, PMID: 34043903 PMC8317550

[ref13] Bouchard M.-A. Target M. Lecours S. Fonagy P. Tremblay L.-M. Schachter A.. (2008). Mentalization in adult attachment narratives: reflective functioning, mental states, and affect elaboration compared. Psychoanal. Psychol. 25, 47–66. doi: 10.1037/0736-9735.25.1.47

[ref14] Brackett M. A. Mayer J. D. Warner R. M. (2004). Emotional intelligence and its relation to everyday behaviour. Personal. Individ. Differ. 36, 1387–1402. doi: 10.1016/S0191-8869(03)00236-8

[ref15] Brody L. R. Hall J. A. (2008). “Gender and emotion in context” in Handbook of emotions. eds. Lewis M. Haviland J. M. Feldmand Barrett L., vol. 3 (New York: Guilford Press), 395–408.

[ref16] Brugnera A. Zarbo C. Compare A. Talia A. Tasca G. A. de Jong K.. (2021). Self-reported reflective functioning mediates the association between attachment insecurity and well-being among psychotherapists. Psychother. Res. 31, 247–257. doi: 10.1080/10503307.2020.1762946, PMID: 32429777

[ref17] Chaplin T. M. (2015). Gender and emotion expression: a developmental contextual perspective. Emot. Rev. 7, 14–21. doi: 10.1177/1754073914544408, PMID: 26089983 PMC4469291

[ref18] Cohen J. (1992). A power primer. Psychol. Bull. 112, 155–159. doi: 10.1037/0033-2909.112.1.155, PMID: 19565683

[ref19] Cologon J. Schweitzer R. D. King R. Nolte T. (2017). Therapist reflective functioning, therapist attachment style and therapist effectiveness. Adm. Policy Ment. Health Ment. Health Serv. Res. 44, 614–625. doi: 10.1007/s10488-017-0790-5, PMID: 28132188

[ref22] Dimitrijević A. Hanak N. Altaras Dimitrijević A. Jolić Marjanović Z. (2018). The Mentalization scale (MentS): a self-report measure for the assessment of Mentalizing capacity. J. Pers. Assess. 100, 268–280. doi: 10.1080/00223891.2017.1310730, PMID: 28436689

[ref23] Đorđević T. Đorđević M. (2019). Recognizing emotions, attachment and mentalization capacity. Int. J. Educ. Psychol. Commun. 9, 7–26.

[ref24] Ensink K. Maheux J. Normandin L. Sabourin S. Diguer L. Berthelot N.. (2013). The impact of mentalization training on the reflective function of novice therapists: a randomized controlled trial. Psychother. Res. 23, 526–538. doi: 10.1080/10503307.2013.800950, PMID: 23964813

[ref25] Field A. (2013). Discovering statistics using IBM SPSS statistics: And sex and drugs and rock 'n' roll. 4th Edn. London: SAGE.

[ref26] Firth N. Barkham M. Kellett S. Saxon D. (2015). Therapist effects and moderators of effectiveness and efficiency in psychological wellbeing practitioners: a multilevel modelling analysis. Behav. Res. Ther. 69, 54–62. doi: 10.1016/j.brat.2015.04.001, PMID: 25880229

[ref27] Fischer-Kern M. Buchheim A. Hörz S. Schuster P. Doering S. Kapusta N. D.. (2010). The relationship between personality organization, reflective functioning, and psychiatric classification in borderline personality disorder. Psychoanal. Psychol. 27, 395–409. doi: 10.1037/a0020862

[ref28] Fischer-Kern M. Doering S. Taubner S. Hörz S. Zimmermann J. Rentrop M.. (2015). Transference-focused psychotherapy for borderline personality disorder: change in reflective function. Br. J. Psychiatry 207, 173–174. doi: 10.1192/bjp.bp.113.143842, PMID: 25999334

[ref29] Fivush R. Bohanek J. G. Zaman W. Grapin S. (2012). Gender differences in adolescents’ autobiographical narratives. J. Cogn. Dev. 13, 295–319. doi: 10.1080/15248372.2011.590787

[ref30] Fivush R. Brotman M. A. Buckner J. P. Goodman S. H. (2000). Gender differences in Parent–child emotion narratives. Sex Roles 42, 233–253. doi: 10.1023/A:1007091207068

[ref31] Fivush R. Haden C. A. (2003). “Creating gender and identity through autobiographical narratives” in Autobiographical memory and the construction of a narrative self: Developmental and cultural perspectives. eds. Fivush R. Haden C. A.. 1st ed (New York: Psychology Press), 149–167.

[ref32] Focquaert F. Steven M. S. Wolford G. L. Colden A. Gazzaniga M. S. (2007). Empathizing and systemizing cognitive traits in the sciences and humanities. Personal. Individ. Differ. 43, 619–625. doi: 10.1016/j.paid.2007.01.004

[ref33] Fonagy P. (2008). “The Mentalization-focused approach to social development” in Mentalization. ed. Busch F. N. (New York: Taylor & Francis Group), 3–56.

[ref34] Fonagy P. Allison E. (2012). “What is mentalization? The concept and its foundations in developmental research” in Minding the child. eds. Midgley N. Vrouva I. (East Sussex: Routledge), 11–34.

[ref35] Fonagy P. Bateman A. W. (2006). Mechanisms of change in mentalization-based treatment of BPD. J. Clin. Psychol. 62, 411–430. doi: 10.1002/jclp.20241, PMID: 16470710

[ref36] Fonagy P. Bateman A. (2019). Handbook of Mentalizing in mental health practice. Washington DC: American Psychiatric Association Publishing.

[ref37] Fonagy P. Gergely G. Jurist E. Target M. (2002). Affect regulation, mentalization, and the development of the self. London: Routledge.

[ref38] Fonagy P. Luyten P. (2009). A developmental, mentalization-based approach to the understanding and treatment of borderline personality disorder. Dev. Psychopathol. 21, 1355–1381. doi: 10.1017/S0954579409990198, PMID: 19825272

[ref39] Fonagy P. Luyten P. (2016). “A multilevel perspective on the development of borderline personality disorder” in Developmental psychopathology: Maladaptation and psychopathology, vol. 3. 3rd Edn. ed. Cicchetti D. (Hoboken: John Wiley & Sons, Inc.), 726–792.

[ref40] Fonagy P. Luyten P. Moulton-Perkins A. Lee Y.-W. Warren F. Howard S.. (2016). Development and validation of a self-report measure of mentalizing: the reflective functioning questionnaire. PLoS One 11:e0158678. doi: 10.1371/journal.pone.0158678, PMID: 27392018 PMC4938585

[ref41] Fonagy P. Target M. (2008). “Attachment, trauma, and psychoanalysis: where psychoanalysis meets neuroscience” in Mind to mind: Infant research, neuroscience, and psychoanalysis. eds. Jurist E. L. Slade A. Bergner S. (New York: Other Press), 15–49.

[ref42] Fonagy P. Target M. Steele H. Steele M. (1998). Reflective-functioning manual, version 5.0, for application to adult attachment interviews. London: University College London, 161–162.

[ref43] George C. Kaplan N. Main M. (1996). Adult Attachment Interview. Berkeley: Department of Psychology, University of California.

[ref44] Goodman G. (2013). Is mentalization a common process factor in transference-focused psychotherapy and dialectical behavior therapy sessions? J. Psychother. Integr. 23, 179–192. doi: 10.1037/a0032354

[ref45] Grysman A. (2018). Gender and gender typicality in autobiographical memory: a replication and extension. Memory 26, 238–250. doi: 10.1080/09658211.2017.1347186, PMID: 28675083

[ref46] Heinonen E. Nissen-Lie H. A. (2020). The professional and personal characteristics of effective psychotherapists: a systematic review. Psychother. Res. 30, 417–432. doi: 10.1080/10503307.2019.1620366, PMID: 31122157

[ref47] Hiirola A. Pirkola S. Karukivi M. Markkula N. Bagby R. M. Joukamaa M.. (2017). An evaluation of the absolute and relative stability of alexithymia over 11years in a Finnish general population. J. Psychosom. Res. 95, 81–87. doi: 10.1016/j.jpsychores.2017.02.007, PMID: 28314554

[ref49] Jańczak M. (2021). Polish adaptation and validation of the Mentalization scale (MentS)—a self-report measure of mentalizing. Psychiatr. Pol. 55, 1257–1274. doi: 10.12740/PP/125383, PMID: 35472226

[ref50] Jessee A. Mangelsdorf S. C. Wong M. S. Schoppe-Sullivan S. J. Brown G. L. (2016). Structure of reflective functioning and adult attachment scales: overlap and distinctions. Attach. Hum. Dev. 18, 176–187. doi: 10.1080/14616734.2015.1132240, PMID: 26754258

[ref51] Johns R. G. Barkham M. Kellett S. Saxon D. (2019). A systematic review of therapist effects: a critical narrative update and refinement to Baldwin and Imel's (2013) review. Clin. Psychol. Rev. 67, 78–93. doi: 10.1016/j.cpr.2018.08.004, PMID: 30442478

[ref52] Kernberg O. F. Diamond D. Yeomans F. E. Clarkin J. F. Levy K. N. (2008). “Mentalization and attachment in borderline patients in transference focused psychotherapy” in Mind to mind: Infant research, neuroscience, and psychoanalysis. eds. Jurist E. L. Slade A. Bergner S. (New York: Other Press), 167–201.

[ref53] Kirk R. E. (1996). Practical significance: a concept whose time has come. Educ. Psychol. Meas. 56, 746–759. doi: 10.1177/0013164496056005002

[ref54] Klasen J. Nolte T. Möller H. Taubner S. (2019). Adverse childhood experiences, attachment representations and mentalizing capacity of psychotherapists in training. Z. Psychosom. Med. Psychother. 65, 353–371. doi: 10.13109/zptm.2019.65.4.353, PMID: 31801442

[ref55] Knox S. Hill C. E. (2021). “Training and supervision in psychotherapy: what we know and where we need to go” in Bergin’s and Garfield’s handbook of psychotherapy and behavior change. eds. Barkham M. Lutz W. Castonguay L. G. (Hoboken: John Wiley & Sons, Inc), 327–349.

[ref56] Köber C. Kuhn M. M. Peters I. Habermas T. (2019). Mentalizing oneself: detecting reflective functioning in life narratives. Attach. Hum. Dev. 21, 313–331. doi: 10.1080/14616734.2018.1473886, PMID: 29768982

[ref57] Luyten P. Campbell C. Allison E. Fonagy P. (2020). The Mentalizing approach to psychopathology: state of the art and future directions. Annu. Rev. Clin. Psychol. 16, 297–325. doi: 10.1146/annurev-clinpsy-071919-015355, PMID: 32023093

[ref58] Luyten P. Fonagy P. (2015). The neurobiology of mentalizing. Personal. Disord. Theory Res. Treat. 6, 366–379. doi: 10.1037/per0000117 26436580

[ref59] Luyten P. Malcorps S. Fonagy P. Ensink K. (2019). “Assessment of Mentalizing” in Handbook of Mentalizing in mental health practice. eds. Bateman A. Fonagy P. (Washington DC: American Psychiatric Association Publishing), 37–63.

[ref60] Müller C. Kaufhold J. Overbeck G. Grabhorn R. (2006). The importance of reflective functioning to the diagnosis of psychic structure. Psychol. Psychother. Theory Res. Pract. 79, 485–494. doi: 10.1348/147608305x68048 17312866

[ref61] Müller S. Wendt L. P. Spitzer C. Masuhr O. Back S. N. Zimmermann J. (2022). A critical evaluation of the reflective functioning questionnaire (RFQ). J. Pers. Assess. 104, 613–627. doi: 10.1080/00223891.2021.1981346, PMID: 34597256

[ref62] Murphy B. A. Lilienfeld S. O. (2019). Are self-report cognitive empathy ratings valid proxies for cognitive empathy ability? Negligible meta-analytic relations with behavioral task performance. Psychol. Assess. 31, 1062–1072. doi: 10.1037/pas0000732, PMID: 31120296

[ref63] Nolte T. Bolling D. Hudac C. Fonagy P. Mayes L. Pelphrey K. (2013). Brain mechanisms underlying the impact of attachment-related stress on social cognition [original research]. Front. Hum. Neurosci. 816, 1–12. doi: 10.3389/fnhum.2013.00816, PMID: 24348364 PMC3841757

[ref65] Pazzagli C. Delvecchio E. Raspa V. Mazzeschi C. Luyten P. (2018). The parental reflective functioning questionnaire in mothers and fathers of school-aged children. J. Child Fam. Stud. 27, 80–90. doi: 10.1007/s10826-017-0856-8

[ref66] Reading R. A. Safran J. D. Origlieri A. Muran J. C. (2019). Investigating therapist reflective functioning, therapeutic process, and outcome. Psychoanal. Psychol. 36, 115–121. doi: 10.1037/pap0000213

[ref67] Richter F. Steinmair D. Löffler-Stastka H. (2021). Construct validity of the Mentalization scale (MentS) within a mixed psychiatric sample [brief research report]. Front. Psychol. 12, 1–9. doi: 10.3389/fpsyg.2021.608214, PMID: 34149501 PMC8210847

[ref68] Rogoff S. Moulton-Perkins A. Warren F. Nolte T. Fonagy P. (2021). ‘Rich’ and ‘poor’ in mentalizing: do expert mentalizers exist? PLoS One 16:e0259030. doi: 10.1371/journal.pone.0259030, PMID: 34695171 PMC8544847

[ref69] Rosso A. M. (2022). Ability emotional intelligence, attachment models, and reflective functioning. Front. Psychol. 13:864446. doi: 10.3389/fpsyg.2022.864446, PMID: 35586239 PMC9108378

[ref70] Saxon D. Firth N. Barkham M. (2017). The relationship between therapist effects and therapy delivery factors: therapy modality, dosage, and non-completion. Adm. Policy Ment. Health Ment. Health Serv. Res. 44, 705–715. doi: 10.1007/s10488-016-0750-5, PMID: 27424106 PMC5550525

[ref71] Stanojević T. S. Radev M. T. Bogdanović A. (2020). From preoccupied attachment to depression: serial mediation model effects on a sample of women. Ljetopis Socijalnog Rada/Annu Social Work 27, 523–542. doi: 10.3935/ljsr.v27i1.334

[ref72] Taubner S. White L. O. Zimmermann J. Fonagy P. Nolte T. (2013). Attachment-related Mentalization moderates the relationship between psychopathic traits and proactive aggression in adolescence. J. Abnorm. Child Psychol. 41, 929–938. doi: 10.1007/s10802-013-9736-x, PMID: 23512713

[ref73] Vrouva I. Target M. Ensink K. (2012). “Measuring mentalization in children and young people” in Minding the child. eds. Midgley N. Vrouva I. (East Sussex: Routledge), 54–76.

[ref74] Wampold B. E. Baldwin S. A. Holtforth M. G. Imel Z. E. (2017). “What characterizes effective therapists?” in How and why are some therapists better than others?: Understanding therapist effects. eds. Castonguay L. G. Hill C. E. (Washington DC: American Psychological Association), 37–53.

